# Boston Hospital, Boston

**Published:** 1906-08-04

**Authors:** A. Cooke Yarborough

**Affiliations:** Secretary.


### EDITOR'S LETTER-BOX.

### BOSTON HOSPITAL, BOSTON.

*To the Editor of* The Hospital.

Sir,—In your last week's issue you give a short account of the new sanitary wing at our hospital, in which you state that the architect was Mr. S. Margason. This is quite correct, but I think it is only fair to say that the plans were drawn from a sketch kindly prepared by Mr. J. G. Oatley, Surveyor to the London Hospital.

I am, yours faithfully,

A. Cooke Yarborough, Secretary.

July 30th, 1906.

